# The incidence of hydrocephalus among patients with and without spinal muscular atrophy (SMA): Results from a US electronic health records study

**DOI:** 10.1186/s13023-021-01822-4

**Published:** 2021-05-07

**Authors:** Emma Viscidi, Nasha Wang, Maneesh Juneja, Ishir Bhan, Claudia Prada, Dayle James, Stacie Lallier, Corinne Makepeace, Karen Laird, Susan Eaton, Anne Dilley, Susan Hall

**Affiliations:** 1 Biogen, Cambridge, MA USA; 2 MJ Analytics Ltd, London, UK; 3 Biogen, Maidenhead, UK

**Keywords:** Spinal muscular atrophy, Hydrocephalus, Nusinersen

## Abstract

**Background:**

The incidence of hydrocephalus in the spinal muscular atrophy (SMA) population relative to the general population is currently unknown. Since the approval of nusinersen, an intrathecally administered drug for SMA, a small number of hydrocephalus cases among nusinersen users have been reported. Currently, the incidence of hydrocephalus in untreated SMA patients is not available, thereby making it difficult to determine if hydrocephalus is a side effect of nusinersen or part of SMA’s natural history. This retrospective, matched cohort study used electronic health records (EHRs) to estimate and compare the incidence of hydrocephalus in both SMA patients and matched non-SMA controls in the time period prior to the approval of nusinersen.

**Methods:**

The U.S. Optum® de-identified EHR database contains records for approximately 100 million persons. The current study period spanned January 1, 2007–December 22, 2016. Patients with SMA were identified by one or more *International Classification of Diseases (ICD)-9* and/or *ICD-10* codes for SMA appearing as primary, admission, or discharge diagnoses, without a pregnancy diagnostic code in the 1-year time before and after the first occurrence of SMA. The first occurrence of SMA defined the index date and non-SMA controls were matched to cases. Incident cases of hydrocephalus were identified with one or more *ICD-9* and/or *ICD-10* code for any type of hydrocephalus following the index date. Hydrocephalus incidence rates per person-months and the incidence rate ratio comparing SMA cases with non-SMA controls were calculated.

**Results:**

There were 5354 SMA cases and an equal number of matched non-SMA controls. Incident hydrocephalus events were identified in 42 SMA cases and 9 non-SMA controls. Hydrocephalus incidence rates per 100,000 person-months were 15.5 (95% CI: 11.2–20.9) among SMA cases and 3.3 (95% CI: 1.5–6.3) among non-SMA controls. The incidence rate ratio was 4.7 (95% CI: 2.4–10.2).

**Conclusions:**

Based on this retrospective analysis utilizing US EHR data, SMA patients had an approximately fourfold increased risk of hydrocephalus compared with non-SMA controls in the era preceding nusinersen treatment. This study may assist in properly evaluating adverse events in nusinersen-treated SMA patients.

**Supplementary Information:**

The online version contains supplementary material available at 10.1186/s13023-021-01822-4.

## Background

Spinal muscular atrophy (SMA) is a neuromuscular disease characterized by the degeneration of motor neurons in the anterior horn of the spinal cord caused by deletions or mutations of the survival motor neuron 1 (*SMN1*) gene [1, 2]. The disease is characterized by progressive muscular weakness with classification based on age of symptom onset and disease severity, which is linked to the number of *SMN2* gene copies present [1–3]. Infants with SMA Type I have symptom onset ≤ 6 months of age, the majority have 2 *SMN2* gene copies, they are unable to sit or roll independently, and eventually have difficulty swallowing and require assisted ventilation [1–3]. Children with SMA Type II have symptom onset between 6–18 months of age, 2–3 *SMN2* gene copies, and can sit unassisted but may not walk independently [1, 2]. Patients with SMA Type III are able to stand and walk unassisted with symptom onset at > 18 months of age, and those with Type IV appear normal until symptoms occur at age > 21 years [1, 2]. Despite being a rare disease and before nusinersen treatment, SMA was the most common monogenetic cause of death in infants [4]. As a genetic disease, new cases of SMA at birth (birth prevalence) are a subset of all cases incident (newly occurring) at conception who survive the fetal period and are born. Among studies from the US, Sweden, and Poland, the birth prevalence of genetically confirmed SMA ranged from 8.5 to 10.3 per 100,000 live births [5–7]. In the United Kingdom (UK), the SMA prevalence in the all-age population is approximately 1.9 cases per 100,000 population [8].

Hydrocephalus is a condition with an abnormal increase in cerebrospinal fluid (CSF) volume with concomitant increase in ventricular size [9, 10]. Hydrocephalus results from an imbalance between the intracranial CSF inflow and outflow. It is caused by obstruction of CSF circulation, due to inadequate absorption of CSF, or less often, by overproduction of the CSF. The excessive volume of CSF causes increased ventricular pressure and ventricular dilatation [11, 12]. Common causes of congenital hydrocephalus include intraventricular hemorrhage and neural tube defects. Other causes include infection, genetic defects, trauma, tumors, and teratogens. Congenital hydrocephalus is often marked by an unusually large head and may include breathing difficulties, poor feeding, and delayed development [10]. The list of symptoms for acquired hydrocephalus is extensive and includes headache, vomiting, drowsiness, and neck pain [10]. In adults, idiopathic normal pressure is the most common form of hydrocephalus and is associated with dementia, gait impairment, and urinary incontinence [13]. Hydrocephalus diagnosed at birth occurs in approximately 78 per 100,000 births in developed countries and 106 per 100,000 births in low- and middle-income countries [14]. The global prevalence varies between age groups, with higher rates observed among children and the elderly: 88, 11, 175, and > 400 per 100,000 individuals among children (≤ 18 years), adults (19–64 years), the elderly (≥ 65 years), and those > 80 years, respectively [14]. The incidence of hydrocephalus in the SMA population in relation to the general population is currently unknown.

Nusinersen, an antisense oligonucleotide, was the first approved treatment for SMA and is administered intrathecally via lumbar puncture. Hydrocephalus has been reported in patients treated with nusinersen in the post-marketing setting [15]. Since the incidence of hydrocephalus in untreated SMA is unknown, it is difficult to determine if hydrocephalus is a side effect of nusinersen treatment or a part of SMA’s natural history. The objective of this study was to conduct a retrospective, matched cohort study of an electronic health record (EHR) database to estimate and compare the incidence of hydrocephalus in SMA patients and matched non-SMA controls during the era preceding nusinersen approval.

## Methods

The US Optum® de-identified EHR database contains records for approximately 100 million persons. The study period was January 1, 2007 through December 22, 2016 (before the approval of nusinersen). SMA cases were identified as patients with one or more *International Classification of Diseases (ICD)-9* and/or *ICD-10* codes for SMA appearing as primary, admission, or discharge diagnoses (Additional file 1). The first observed code defined the SMA index date. Females with a pregnancy record (based on *ICD-9* and/or *ICD-10* diagnostic codes) in the 1-year time period before or after the SMA index date were excluded to reduce the risk of erroneously classifying women who had babies diagnosed with SMA. It is possible that an SMA diagnostic code for an infant was included in the medical record of the mother around the time of the pregnancy or birth.

**Fig. 1:**
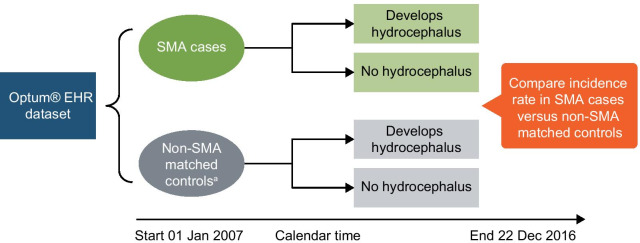
Matched cohort study design of the incidence of hydrocephalus among SMA cases and non-SMA controls. *EHR* electronic health record, *SMA* spinal muscular atrophy. ^a^Non-SMA matched controls matched to SMA cases on birth year, sex, and index date

A set of non-SMA controls were matched 1:1 to cases on birth year and sex, and the assumed the index date of the matched case (Fig. 1). Incident cases of hydrocephalus were identified with one or more *ICD-9* and/or *ICD-10* codes for any type of hydrocephalus following the index date (Additional file 1). Hydrocephalus incidence rate (IR) per person-months (PMs) and 95% confidence intervals (CIs), as well as the incidence rate ratio (IRR) comparing SMA cases to non-SMA controls, were calculated.

## Results

**Fig. 2:**
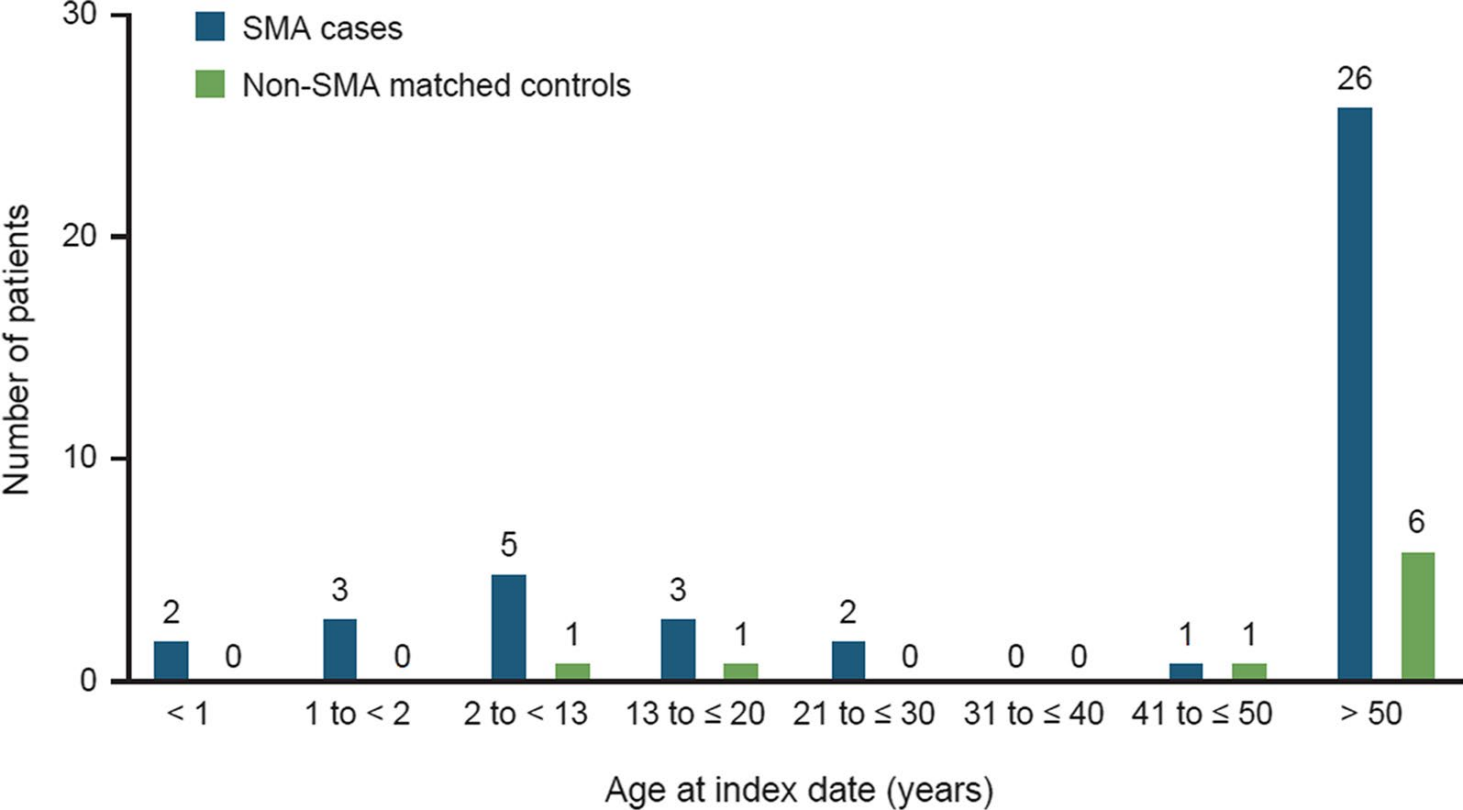
Age at index date among SMA cases (n = 42) and non-SMA matched controls (n = 9) with hydrocephalus. Index date = date of first SMA diagnostic code for cases and a matched date for non-SMA controls. *SMA* spinal muscular atrophy

During the 10-year study period, 5354 SMA cases and 5354 matched non-SMA controls were identified and included in the analyses. The demographics are presented in Table 1. The mean age of the SMA cases was 45 years (range: 0–86 years) and approximately half (53%) of the SMA cases were male. The mean follow-up time in the US Optum® database for the SMA cases was 90.6 months (7.6 years [SD, 3.9 years]). There were 42 incident hydrocephalus events among the SMA cases and 9 incident hydrocephalus events among non-SMA controls. In the SMA cases, the most frequent type was obstructive hydrocephalus (37.5% of all hydrocephalus diagnostic codes; Additional file 2). In the non-SMA control group, the most frequent type was idiopathic normal pressure hydrocephalus (60% of all hydrocephalus diagnostic codes). Among the 42 SMA patients with hydrocephalus, the mean age was 46.9 years and 57% were male. The mean age of the nine non-SMA controls with hydrocephalus was slightly older at 59.7 years, and 67% were male. The greatest number of hydrocephalus cases occurred in patients older than 50 years of age, among both SMA cases and non-SMA controls (Fig. 2). There were 5 cases of hydrocephalus in children younger than age 2 years, all of whom were SMA patients.

**Table 1:** Patient demographics

Category	SMA cases (N = 5354)	SMA cases who developed hydrocephalus (N = 42)	Non-SMA matched controls (N = 5354)	Non-SMA matched controls who developed hydrocephalus (N = 9)
Age at index date (years)				
Mean	45.0	46.9	45.0	59.7
Median	45	57	45	70
Min, max	0, 86	0, 86	0, 86	8, 85
SD	23.65	30.59	23.65	27.99
Age (years) at index date categories (n, %)				
0 to < 1	95 (1.8)	2 (4.8)	95 (1.8)	0 (0.0)
1 to < 2	114 (2.1)	3 (7.1)	114 (2.1)	0 (0.0)
2 to < 13	442 (8.3)	5 (12)	442 (8.3)	1 (11.0)
13 to ≤ 20	326 (6.1)	3 (7.1)	326 (6.1)	1 (11.0)
21 to ≤ 30	436 (8.1)	2 (4.8)	436 (8.1)	0 (0.0)
31 to ≤ 40	926 (17.0)	0 (0.0)	926 (17.0)	0 (0.0)
41 to ≤ 50	653 (12.0)	1 (2.4)	653 (12.0)	1 (11.0)
> 50	2362 (44.0)	26 (62.0)	2362 (44.0)	6 (67.0)
Sex (n, %)				
Female	2521 (47.0)	18 (43.0)	2521 (47.0)	3 (33.0)
Male	2833 (53.0)	24 (57.0)	2833 (53.0)	6 (67.0)
Time in database (months)				
Mean	90.6	96.5	90.6	136.4
Median	93	107.5	93	159
SD	46.65	54.66	46.65	42.69

*SD* standard deviation, *SMA* spinal muscular atrophy

The overall incidence of hydrocephalus was greater in SMA cases than non-SMA controls (Table 2). The IR of hydrocephalus was 15.5 per 100,000 PMs (95% CI: 11.2–20.9) among SMA cases, compared with 3.3 per 100,000 PMs (95% CI: 1.5–6.3) among non-SMA controls (Table 2). The IRR between SMA cases and non-SMA controls was 4.7 (95% CI: 2.4–10.2). The IR for hydrocephalus differed by sex and age at SMA index date. The IR was higher in male than female SMA cases (17.0 [95% CI: 10.9–25.3] vs. 13.9 [95% CI: 8.2–21.9]). Similarly, the IR was greater in males than females in the non-SMA control group (4.2 [95% CI: 1.6–9.2] vs. 2.3 [95% CI: 0.5–6.7]). The IRR between SMA cases and non-SMA controls was 6.0 (95% CI: 1.9–25.7) for females and 4.0 (95% CI: 1.7–10.8) for males. Among the SMA cases, the IR was higher in younger cases; 50.7 (95% CI: 6.1–183.3) and 55.5 (95% CI: 11.4–162.1) in the < 1-year and the 1- to < 2-year age groups, respectively. In the non-SMA control group, the IR was higher in ages 13 to ≤ 20 years (5.5 [95% CI: 0.1–30.7]) and the > 50-year (5.0 [95% CI: 1.8–10.9]) age groups.

**Table 2:** Hydrocephalus incidence rates

	Rate per 100,000 PMs among SMA cases (N = 5354) (IR [95% CI])	Rate per 100,000 PMs among non-SMA matched controls (N = 5354) (IR [95% CI])	Incidence rate ratio^a^ (95% CI)
Incident hydrocephalus events	42	9	
Overall incidence rate	15.5 (11.2–20.9)	3.3 (1.5–6.3)	4.7 (2.4–10.2)
Incidence rate by sex			
Female	13.9 (8.2–21.9)	2.3 (0.5–6.7)	6.0 (1.9–25.7)
Male	17.0 (10.9–25.3)	4.2 (1.6–9.2)	4.0 (1.7–10.8)
Incidence rate by age at index date (years)			
< 1	50.7 (6.1–183.3)	0.00	N/C
1 to < 2	55.5 (11.4–162.1)	0.00	N/C
2 to < 13	17.3 (5.6–40.4)	3.4 (0.1–19.1)	5.1 (0.7–120.1)
13 to ≤ 20	16.6 (3.4–48.5)	5.5 (0.1–30.7)	3.0 (0.3–79.3)
21 to ≤ 30	9.8 (1.2–35.5)	0.00	N/C
31 to ≤ 40	0.00	0.00	N/C
41 to ≤ 50	2.8 (0.1–15.9)	2.8 (0.1–15.9)	1.0 (0.0–39.0)
> 50	21.7 (14.2–31.8)	5.0 (1.8–10.9)	4.4 (1.9–11.6)

*CI* confidence interval, *IR* incidence rate, *IRR* incidence rate ratio, *N/C* not calculable, *PM* person-month, *SMA* spinal muscular atrophy

^a^95% CI for IRR not calculated due to small sample size

In SMA patients, 12.5% of diagnostic codes were communicating hydrocephalus (10 of the 80 total diagnostic codes for hydrocephalus) as compared to 20% in the control group (3 of 15 total diagnostic codes) (Additional file 2). Additionally, 15% of the hydrocephalus diagnostic codes in the SMA group were classified as unspecified hydrocephalus (vs. 6.7% in controls). The incidence rate for communicating hydrocephalus was 3.3 (95% CI, 1.51–6.27) in the SMA group and 0.73 (95% CI, 0.09–2.7) in the control group (data not presented in tables). The relative risk comparing communicating hydrocephalus in cases vs. controls was 4.51 (95% CI, 1.07–30.60), indicating that communicating hydrocephalus was significantly more common in SMA patients than controls.

## Discussion

Based on this retrospective analysis using real-world EHR data, SMA patients had a fourfold increased risk of hydrocephalus compared with matched non-SMA controls. The increased risk of hydrocephalus in SMA was observed in both males and females and across the various ages at SMA index date. This study, conducted using data before the approval of nusinersen, suggests that the SMA disease state may increase the risk of hydrocephalus; however, further research is needed to determine any causal association between SMA and hydrocephalus. Causality notwithstanding, these data provide a new understanding of the natural history of SMA and may provide context for newly observed hydrocephalus events among SMA patients.

The preponderance of hydrocephalus in older SMA patients, which we can safely assume are Type II–III patients, was an unexpected finding. However, the incidence of hydrocephalus observed among > 50-year-old SMA patients is similar to the higher incidence of hydrocephalus among the elderly in the general population [14]. The global prevalence of hydrocephalus is substantially higher among the elderly and, in particular, those > 80 years of age [14]. Among SMA patients, the most frequent type was obstructive hydrocephalus, which may be indicative of the systemic nature of SMA. In the general population, there is no identifiable cause for idiopathic normal pressure hydrocephalus in older patients, whereas symptomatic hydrocephalus is typically linked to brain infection, injury, or hemorrhage [10, 13]. In addition, the incidence of hydrocephalus was highest in the younger age groups of SMA patients (younger than 2 years), whereas in controls, the incidence rate was highest in ages 13 to ≤ 20 years and > 50 years.

The increased risk of hydrocephalus in SMA patients may be in part due to surveillance bias. SMA patients may be more likely to receive routine magnetic resonance imaging (MRI), leading to the detection of asymptomatic hydrocephalus. Adult patients, in particular, may be more likely to have asymptomatic hydrocephalus [13]. It is unlikely that the majority of hydrocephalus cases were asymptomatic, and thus, it is not expected that this would account entirely for the increased number of cases of hydrocephalus in SMA patients. Future studies may be able to address this type of potential confounding by adjusting for comorbid diagnoses and surveillance bias when comparing hydrocephalus in cases and controls.

The strengths of this study include the large population-based sample of more than 5000 SMA patients and the long follow-up time (7.6-year study period [SD, 3.8 years]), which allowed for the capture of incident medical events. Additionally, the wide range of ages covered in this study is beneficial given that nusinersen treatment among adults has increased since its approval, and we have limited adult data in our clinical trials. The limitations of this analysis include a lack of information on the specific SMA type, the reliance on diagnostic codes to define disease states, the lack of information on the cause of hydrocephalus, and the uncertainty as to whether the observed SMA index date truly represents the SMA diagnosis date. In this study, approximately 4% of SMA patients were younger than 2 years at index date, whereas the majority were older than 2 years at index date, which is markedly different than the US prevalence by age. In the US, the estimated percentages of SMA patients by SMA type are: 17% Type I patients (symptom onset < 6 months of age), 42% Type II (6–18 months of age), and 41% Type III (> 18 months of age) [7]. The older ages of the SMA patients in this study are likely due to survival bias in the pre-treatment era, and that for many patients, the SMA index date was not the actual diagnosis or symptom onset date, but instead the first SMA diagnostic code appearing in their medical record in the database.

A few case studies have reported on hydrocephalus in SMA patients unrelated to nusinersen treatment, with the majority observed in patients with the severe phenotypes, SMA Type 0–I [16–19]. The earliest case was reported in 2014, before the approval of nusinersen, in a patient with SMA Type I requiring ventilation soon after birth; the patient was diagnosed at 5 months with hydrocephalus due to Blake’s pouch cyst, an abnormal embryogenic regression that causes ventricular enlargement [16]. Since Blake’s pouch is a structural malformation, the hydrocephalus diagnosis in these patients is unlikely due to nusinersen treatment. A 2019 case study from Brazil reported two children (ages 1 and 3 years) with SMA Type 0, both with persistent Blake’s pouch cyst and requiring ventilation support [18]. This report did not indicate if the children were treated with nusinersen, yet it is unlikely that the hydrocephalus was related to nusinersen since it was not yet commercially available in Brazil [18]. In 2020, one case report described a patient diagnosed with SMA Type I soon after birth, requiring ventilation at 2 months who was subsequently diagnosed with hydrocephalus due to Blake’s pouch cyst [17]. The patient received a tracheostomy and ventriculoperitoneal shunt (VPS) at 4 months and did not start nusinersen treatment until age 7 years via VPS without exacerbation of the hydrocephalus [17]. A recent report published in 2020 described a female infant diagnosed with SMA Type I at age 2 months with communicating hydrocephalus who died at age 5 months due to secondary to aspiration pneumonia. The patient did not receive nusinersen treatment [19].

It has been speculated that SMA may present with hydrocephalus involving the rare condition of Dandy-Walker syndrome (DWS) which is a continuum of congenital defects, including Blake’s pouch cyst [20]. DWS is rare (1 in 25,000), with postnatal hydrocephalus occurring in approximately 80% of cases [21]. Congenital heart defects and malformations of the palate, vertebrae, and genitalia are also associated with DWS [21]. A few single case reports have proposed a possible link between SMA and DWS; however, in patients of severe and complex congenital malformations without genetic confirmation, it may be difficult to isolate a definitive diagnosis [19, 20, 22, 23]. Further research is necessary to determine if a genetic link exists between DWS, specifically Blake’s pouch cyst, and SMA disease, which may contribute to the incidence of hydrocephalus observed in SMA patients aged 0–2 years.

In 2018, Biogen issued a “Dear Healthcare Professional” letter advising clinicians to monitor for symptoms of hydrocephalus after five SMA patients treated with nusinersen were identified with communicating hydrocephalus [24]. Communicating hydrocephalus is caused by an impaired absorption of CSF with no apparent obstruction of CSF flow [10, 25]. Of the five SMA patients in this report, four were SMA Type I patients, ages 4 months to 3 years, who received 2–4 doses of nusinersen. The fifth patient was an adult female with scoliosis, and no details were reported about her age at symptom onset or number of doses. Since the 2018 report, three male infants with SMA Type I and treated with nusinersen were diagnosed with hydrocephalus [26–28]. Two infants initiated nusinersen at age 2.5 months and age 4 months and were diagnosed with communicating hydrocephalus after 3 and 4 doses, respectively [26, 28]. The third infant was diagnosed with SMA Type I at age 2 months, required ventilator and feeding support at 17 months, and started nusinersen at age 6 years [27]. After 18 months of treatment, the patient was diagnosed with triventricular hydrocephalus [27]. It should be noted that two of the patients with MRI prior to their SMA diagnosis showed moderate ventricular dilation, suggesting that the development of hydrocephalus may have started prior to the initiation of nusinersen treatment [27, 28]. Despite these case reports, there have been no reports of hydrocephalus in the index nusinersen trials of pre-symptomatic infants (most likely to develop SMA Type I or II), infantile-onset (most likely to develop Type I), and later-onset (most likely to develop Type II or III) SMA participants [24, 29–32]. Nor have there been any reports of hydrocephalus in the follow-up interim analyses of the NURTURE study or the open-label SHINE extension trial with up to 6.8 years of nusinersen treatment [33–36].

Future studies on this topic should address the specific type of hydrocephalus most common in SMA patients, and whether the incidence of this type (or types) is greater in SMA patients compared to matched controls. In addition, in the era of disease-modifying therapies, it would be useful to determine if there is a temporal relation between the occurrence of hydrocephalus and treatment with nusinersen or other therapies.

## Conclusions

In this real-world analysis of SMA patients and matched non-SMA controls, conducted in the time period before nusinersen approval, the incidence of hydrocephalus was 4 times greater in SMA patients than non-SMA controls. The increased incidence rates were observed across the different age groups, with the highest found among the youngest and the oldest age groups. Future studies on this topic are needed to determine whether hydrocephalus is part of the natural history of SMA in some patients, and whether there is a causal association between SMA and hydrocephalus. These findings are important for the optimization of care and treatment of patients with SMA and when conducting and interpreting post-marketing safety studies.

## Supplementary Information

**Additional File 1.**. Table of ICD codes.

**Additional File 2.** Table of frequency of incident hydrocephalus *ICD* codes.

## Data Availability

The dataset supporting the conclusions of this article are available in the U.S. Optum® EHR repository and hyperlink to dataset(s) in https://www.optum.com/business/solutions/government/federal/data-analytics-federal/clinical-data.html#.

## Abbreviations

CI: Confidence interval
CSF: Cerebrospinal fluid
DWS: Dandy–Walker syndrome
EHR: Electronic health record
*ICD*: *International Classification of Diseases*
IR: Incidence rate
IRR: Incidence rate ratio
PM: Person-month
SD: Standard deviation
SMA: Spinal muscular atrophy
*SMN1*: Survival motor neuron 1
UK: United Kingdom
US: United States
VPS: Ventriculoperitoneal shunt
