# Supplementary material for: The incidence of hydrocephalus among patients with and without spinal muscular atrophy (SMA): Results from a US electronic health records study
Source: Orphanet J Rare Dis. 2021 May 7;16:207. doi: 10.1186/s13023-021-01822-4 (PMC8105953; doi:10.1186/s13023-021-01822-4)
Supplement: Supplementary file 1 — Additional File 1.. Table of ICD codes. [file 13023_2021_1822_MOESM1_ESM.docx]

**Additional file 1.** *ICD* codes

| ***ICD* codes** | **Label** |
| --- | --- |
| **Spinal muscular atrophy** | |
| 335.0 | Infantile spinal muscular atrophy |
| 335.10 | Spinal muscular atrophy |
| 335.11 | Juvenile spinal muscular atrophy |
| 335.19 | Adult spinal muscular atrophy |
| G12.0 | Infantile spinal muscular atrophy |
| G12.1 | Other inherited spinal muscular atrophy |
| G12.8 | Other spinal muscular atrophies and related syndromes |
| G12.9 | Spinal muscular atrophy, unspecified |
|  |  |
| **Hydrocephalus** | |
| 7423 | Congenital hydrocephalus |
| 3313 | Communicating hydrocephalus |
| 3314 | Obstructive hydrocephalus |
| 3315 | Idiopathic normal pressure hydrocephalus |
| 6536 | Hydrocephalic fetus causing disproportion |
| 65360 | Hydrocephalic fetus causing disproportion unspecified as to episode of care |
| 65361 | Hydrocephalic fetus causing disproportion delivered |
| 65363 | Hydrocephalic fetus causing disproportion antepartum |
| 7410 | Spina bifida with hydrocephalus |
| 74100 | Spina bifida with hydrocephalus unspecified |
| 74101 | Spina bifida cervical region with hydrocephalus |
| 74102 | Spina bifida dorsal (thoracic) region with hydrocephalus |
| 74103 | Spina bifida lumbar region with hydrocephalus |
| G91 | Hydrocephalus |
| G910 | Communicating hydrocephalus |
| G911 | Obstructive hydrocephalus |
| G912 | Idiopathic normal pressure hydrocephalus |
| G913 | Post-traumatic hydrocephalus, unspecified |
| G914 | Hydrocephalus in diseases classified elsewhere |
| G918 | Other hydrocephalus |
| G919 | Hydrocephalus, unspecified |
| Q03 | Congenital hydrocephalus |
| Q038 | Other congenital hydrocephalus |
| Q039 | Congenital hydrocephalus, unspecified |
| Q050 | Cervical spina bifida with hydrocephalus |
| Q051 | Thoracic spina bifida with hydrocephalus |
| Q052 | Lumbar spina bifida with hydrocephalus |
| Q053 | Sacral spina bifida with hydrocephalus |
| Q054 | Unspecified spina bifida with hydrocephalus |
| Q0702 | Arnold-Chiari syndrome with hydrocephalus |
| Q0703 | Arnold-Chiari syndrome with spina bifida and hydrocephalus |
| O336 | Maternal care for disproportion due to hydrocephalic fetus |
| O336XX0 | Maternal care for disproportion due to hydrocephalic fetus, unspecified |
| O336XX1 | Maternal care for disproportion due to hydrocephalic fetus, fetus 1 |
| O336XX2 | Maternal care for disproportion due to hydrocephalic fetus, fetus 2 |
| O336XX3 | Maternal care for disproportion due to hydrocephalic fetus, fetus 3 |
| O336XX4 | Maternal care for disproportion due to hydrocephalic fetus, fetus 4 |
| O336XX5 | Maternal care for disproportion due to hydrocephalic fetus, fetus 5 |
| O336XX9 | Maternal care for disproportion due to hydrocephalic fetus, other |

*ICD International Classification of Diseases.*
