# Supplementary material for: The incidence of hydrocephalus among patients with and without spinal muscular atrophy (SMA): Results from a US electronic health records study
Source: Orphanet J Rare Dis. 2021 May 7;16:207. doi: 10.1186/s13023-021-01822-4 (PMC8105953; doi:10.1186/s13023-021-01822-4)
Supplement: Supplementary file 2 — Additional File 2. Table of frequency of incident hydrocephalus ICD codes. [file 13023_2021_1822_MOESM2_ESM.docx]

**Additional file 2.** Frequency of incident hydrocephalus *ICD* codes

| ***ICD* codes** | **Label** | **Hydrocephalus *ICD* codes reported in the SMA group**  **(N=80)**  **(n, %)** | **Hydrocephalus *ICD* codes reported in the non-SMA matched controls  (N=15)**  **(n, %)** |
| --- | --- | --- | --- |
| G919 | Hydrocephalus, unspecified | 12 (15.0) | 1 (6.7) |
| Q038/Q039/7423 | Congenital hydrocephalus | 8 (10.0) | 0 |
| 3313/ G910 | Communicating hydrocephalus | 10 (12.5) | 3 (20.0) |
| 3314/G911 | Obstructive hydrocephalus | 30 (37.5) | 2 (13.3) |
| 3315/G912 | Idiopathic normal pressure hydrocephalus | 15 (18.8) | 9 (60.0) |
| 74100/Q054 | Spina bifida w hydrocephalus, unspecified | 3 (3.8) | 0 |
| 74102 | Spina bifida dorsal (thoracic) region with hydrocephalus | 1 (1.3) | 0 |
| G913 | Post-traumatic hydrocephalus, unspecified | 1 (1.3) | 0 |

Patients may have had one or more *ICD* codes listed for hydrocephalus.

*ICD International Classification of Diseases.*
